# Radical Gas-Based DNA Decontamination for Ultra-Sensitive Molecular Experiments

**DOI:** 10.1264/jsme2.ME12061

**Published:** 2012-04-18

**Authors:** Yuki Morono, Katsuhiro Yamamoto, Fumio Inagaki

**Affiliations:** 1 Geomicrobiology Group, Kochi Institute for Core Sample Research, Japan Agency for Marine-Earth Science and Technology (JAMSTEC), Monobe B200, Nankoku, Kochi 783–8502, Japan; 2 Geobio-Engineering and Technology Group, Submarine Resources Research Project, JAMSTEC, Monobe B200, Nankoku, Kochi 783–8502, Japan; 3 Department of Materials Science and Engineering, Graduate School of Engineering, Nagoya Institute of Technology, Gokiso-cho, Showa-ku, Nagoya 466–8555, Japan

**Keywords:** DNA decontamination, methanol radical, ultra-sensitive molecular approach

## Abstract

In this study, we tested a radical gas-based decontamination technique to prevent possible DNA contamination by the air and/or equipment used in molecular experiments. We prepared 10^4^ molecules of model DNA contaminant and placed the dried DNA into test tubes, which were then exposed to radical gas. Quantitative PCR analysis showed that, even after a short exposure time of 30 minutes, 99.54% of the model DNA contaminant was effectively decomposed to undetectable levels. Our results demonstrate that the radical gas-based treatment is a useful method for eliminating potential DNA contaminant in ultra-sensitive molecular experiments.

Extremely sensitive and small-scale molecular approaches have become powerful tools in the study of small amounts of materials in microbial ecology. Such ultra-sensitive molecular approaches include, for example, single-copy detection of target genes by PCR and single-cell genome amplification with multiple displacement amplification (MDA) (7, 12, 13). In these experiments using powerful polymerases, the contamination potential of laboratory-derived DNA molecules can hamper high-quality results, since even very low levels of contamination can lead to false-positive amplification (2). To avoid a loss of reliability in the obtained amplified product, various DNA decontamination methods have been developed for ultra-sensitive molecular experiments. Previously proposed approaches for DNA decontamination include UV (14, 16) and gamma irradiation (6), chemical (8) and exonuclease ([4] and references therein) treatments, and autoclaving; however, the development of decontamination techniques for analytical equipment (*e.g.*, pipettes, stands and other tools for handling), which are very important components, has remained difficult. In addition, currently available decontamination methods often utilize oxidative reagents and/or UV, which may damage tools and other materials.

In this report, we demonstrate a radical gas-based decontamination method. Methanol-derived radical gas (Seki, H., T. Kuwabara, M. Kubo, and S. Okada. Japan Patent Kokai, P2005-130993A) is a mixture of gaseous methanol and reactive radical species generated through interactions between gaseous methanol and a catalyst. Although the exact composition of radical species in methanol-derived radical gas remains unknown, the radical species in the gas can induce the breakage of DNA strands (3). Treatment time is controllable by the supply of methanol to the gas generator (URM-50-1 [WIZSYSTEMS Co., LTD. and SEALIVE Inc. Japan Patent 4292234]; URM, Nagoya, Japan). To test the effectiveness of the radical-based decontamination approach, we prepared a series of experiments in order to confirm the follows: (i) efficiency and time course of model DNA contaminant degradation; (ii) quantity and quality of degradation, and its efficiency; and (iii) remnants of any gas components that may inhibit post-exposure PCR amplification. The exposure experiment was performed using the URM-50-1 gas generator that has been deployed at the JAMSTEC Geomicrobiology Lab at Kochi after its purchase.

The model contaminant DNA used in this study was fragments of the 16S rRNA gene of *Pyrococcus horikoshii* OT3^T^ JCM9974 (9) amplified by PCR with Arc21F (5) and Univ1490R (10). The quantity and quality of the PCR fragments was determined fluorometrically with a Qubit fluorometer (Life Technologies, Carlsbad, CA, USA) and electrophoresis. The number of molecules was calculated based on the size of the PCR fragments. Samples (10 μL) of known concentration (1×10^3^ molecules per μL of water) of the test contaminant DNA were placed in 100-μL PCR tubes (Life Technologies) and dried under a vacuum using SpeedVac (Thermo Fisher Scientific, Waltham, MA, USA). Eight replicate samples were exposed to methanol radical gas in a vinyl glove chamber (COY Lab Products, Grass Lake, MI, USA) with custom gas introducing valve. The formation of methanol-derived radical gas was continued for 15 minutes and the test samples were exposed to the gas for 0.5, 1, 2 or 4 hours. As methanol radical gas might contain gaseous methanol and formaldehyde, PCR tubes with contaminant DNA in water (wet control A), PCR tubes with pure water (wet control B), and PCR tubes without DNA (dry control) were exposed as control samples to examine the water-soluble and surface-adsorbed gas component and its effect on PCR amplification. After exposure, samples were retrieved and capped, and were then transferred to the molecular biology lab for subsequent analysis.

The amount of DNA after radical gas treatment was quantified by real-time PCR (StepOnePlus; Life Technologies) by adding 10 μL PCR mixture containing 5 μL of 2× PCR mixture from a Dimer eraser (Takara Bio, Otsu, Japan) and 5 μL water. For amplification, the primer set ARC806F (15) and ARC958R (5) was used, and thermal conditions were 95°C for 30 s for initial denaturation, followed by up to 50 cycles of 95°C for 5 s, 55°C for 30 s and 72°C for 30 s. Standard curves for archaeal 16S rRNA genes were obtained using genomic DNA from *P. horikoshii* OT3^T^ JCM9974 (9) (R^2^=0.995, amplification efficiency was 1.71 [85%]). Since we did not see any amplification after 45 PCR cycles which corresponds to less than 1 copy of template DNA per tube, we considered this (1 copy) as a lower limit of detection for this study. After amplification, melting curve analysis was performed in order to examine the amplified product (data not shown).

Fig. 1 shows the quantification results for the model contaminated DNA. The initial amount of DNA was 1×10^4^ molecules per tube. After exposure for 0.5 h, more than 99.54% of the added DNA was found to be undetectable by real-time PCR. When the exposure time was increased to 4 h, the added DNA went below the detection limit by real time PCR.

We also tested PCR amplification with wet controls with and without model DNA contaminant. No amplifications were observed both in wet control A (water with DNA) and wet control B (pure water only). In the other experiment adding 1 × 10^3^ archaeal 16S rRNA gene fragments to wet control B after exposure to methanol radical gas, no amplifications occurred in the PCR reaction. These results indicate that there was inhibition of PCR enzyme in the wet controls. The water-soluble gas component that inhibited PCR in wet controls was analyzed by ^1^H-NMR (AVANCE200 Bruker) by exposing deuterium oxide to methanol-derived radical gas (wet control C). The main component in the sample was methanol at a concentration of 0.03 wt%. We also detected trace amounts of formic acid in heavy water (^1^H-NMR peak at 8.2 ppm; ***H***-COOH); however, the concentration was very low (<1×10^−5^ wt%) and quantitative data could not be retrieved. None of the other compounds were detected in the analysis; therefore, we attributed the failure of PCR amplification to these water-soluble gas components, and hence methanol radical gas was found not to be applicable to the decomposition of DNA contaminants in the liquid.

We then examined the effect(s) of remnant(s) of methanol radical gas on the dry surface, by adding PCR mixture containing 1×10^3^ archaeal 16S rRNA gene fragments (Fig. 1B) to the tubes that had already been exposed to methanol radical gas. The quantification data showed a small decrease (within the error range) in the quantified number of detected DNA molecules. No detectable time-dependent decreases in the quantity of DNA were observed. Thus, we concluded that the effects of the remnant gas component on PCR enzymatic activity were almost negligible on dry surfaces.

The integrity of DNA contaminant after gas treatment was examined using a microfluidic electrophoresis system (Experion automated electrophoresis system; BioRad Laboratories, Hercules, CA, USA). Ten nanogram of PCR fragments of the 16S rRNA gene of *Pyrococcus horikoshii* was placed in 100-μL PCR tubes, dried, and exposed to methanol radical gas as shown above. Then the remaining DNA was dissolved in 10 μL of water and 1 μL of it was analyzed with DNA-analyzing chip (Experion DNA 12K Chip, BioRad Laboratories). The result shown in Fig. 2A–E demonstrates that there was no detectable peak after exposure of 0.5 h. Since DNA chip only detects double-stranded DNA, we did another analysis with RNA-analyzing chip (Experion RNA HighSens Chip, BioRad Laboratories) and similarly observed no detectable peak after 0.5 h of the exposure (Fig. 2F–I). The double peak on Fig. 2J, which was seen in the analysis of heat-denatured, non-treated DNA, is most likely due to secondary structure formation by analyzed DNA. These results confirm that single- and double-stranded DNA was degraded into small nucleotides (shorter than 50 bp) by radical gas treatment, and this supports the failure of PCR amplification of treated DNA (Fig. 1).

Ultrasensitive detection and amplification approaches have enabled us to open a new field of molecular microbial ecology, targeting low-level or specific biomass and physiologically uncharacterized single cells (1, 11). To apply such ultrasensitive techniques to the study of microbial ecology, the preparation of a cleaner environment, equipment, and reagent is a crucial issue to be addressed. For the decontamination of experimental equipments (*e.g.*, laser micro-dissection, optical tweezers, flow cytometric and other cell sorters, automated pipetting devices, and other tools that are used for ultra-sensitive molecular approaches), currently available techniques are inapplicable because some equipment might be large and complicated in structure, and contain metal and plastic parts that are sensitive to acids and oxidative agents. UV treatment is one of the most common methods of decontamination; however, as it will destroy plastic after repeated use, it is only effective for exposed surfaces with a high-energy dose and not inside a complicated instrument, and it sometimes results in the failure to eliminate amplifiable DNA fragments completely (4). Gamma irradiation is superior in the treatment of complicated instruments and induces double-strand and single-strand breakage of DNA, although the effectiveness to eliminate small DNA fragments is questionable (4). Chemicals (detergents, hypochlorite, etc.) often rely on water (*i.e.,* vapor or mist) for their delivery, which places limitations on penetrating complicated structures. Also, surface-attaching droplets of water may cause damage to electric circuits and, even worse, may increase the corrosion of metals by acidic or oxidative chemicals in the droplets. In addition, autoclaving is not applicable to electric instruments.

In this study, we demonstrated that methanol radical gas nearly completely destroyed the applied model contaminant DNA on dry surfaces within several hours. Moreover, almost no detectable residual components were observed after treatment. In addition to its effectiveness for DNA decomposition, the gaseous nature of methanol radical gas has advantages in its penetration and the gas without water or corrosive chemicals enables the decontamination of electronic equipment. Although this methanol radical gas cannot be applied to liquid material because of the existence of water-soluble compounds, the decontamination of dry surfaces is applicable to many targets, even to a whole room equipped with electronic instruments. In our trial to expose a heat block to methanol radical gas for hours while it was running, we did not see any visible impact on its function. Conclusively, the present methanol radical-based decontamination approach is highly useful in basic cleaning procedures for various laboratory equipment used in ultra-sensitive molecular experiments.

## Figures and Tables

**Fig. 1:**
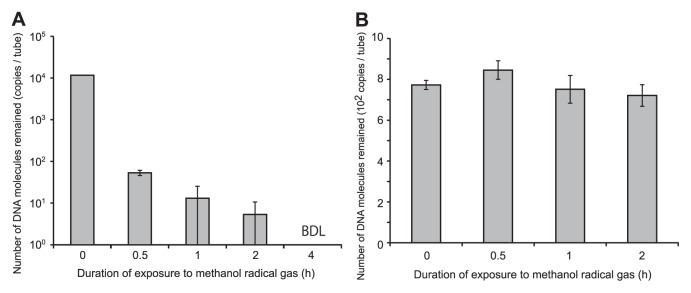
Experimental assessment of the methanol radical gas decontamination method. (**A**) Decomposition of model contaminant DNA with methanol radical gas treatment, as measured by real-time PCR analysis. Each bar shows mean DNA copy number from 8 independent reactions. BDL: below detection limit. (**B**) Effect of remnant gas component on surface of test tubes. After exposure to methanol radical gas for the respective duration, PCR mix containing approximately 1×10^3^ molecules of the template DNA was added and analyzed by realtime PCR.

**Fig. 2:**
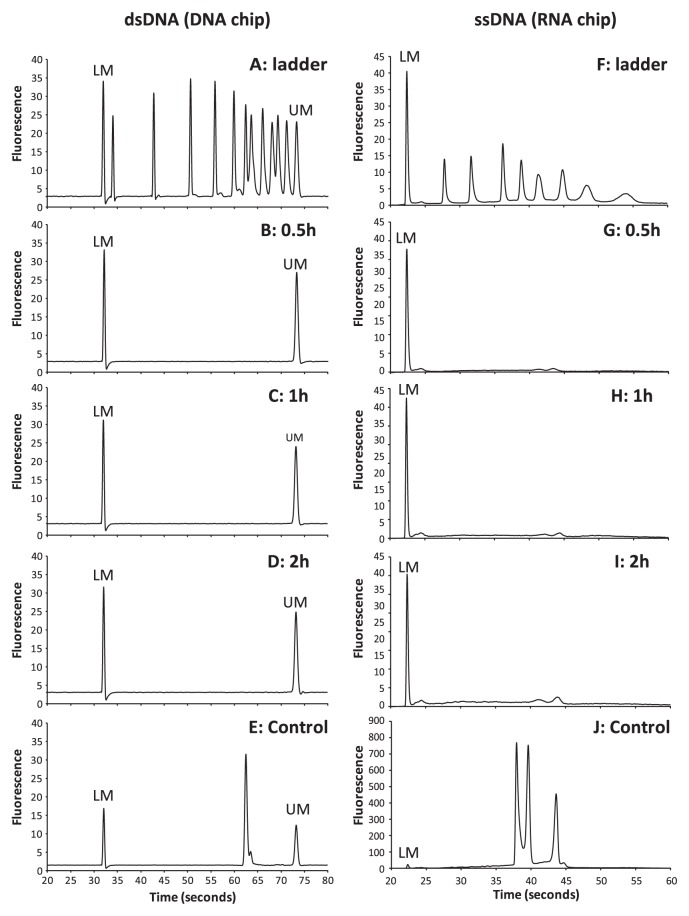
Integrity of methanol radical gas-treated DNA confirmed by microfluidic electrophoresis. DNA- and RNA-analyzing chips (Experion DNA 12K and Experion RNA HighSens Chips, respectively) were used to examine non-degraded double- (**A–E**) and single- (**F–J**) stranded DNA. **A** and **F** show the analysis results for the DNA ladder (100–10380 bp) and RNA ladder (200–6,000 bp), respectively. Analysis results for DNA samples treated with methanol radical gas for 0.5 h (**B**, **G**), 1 h (**C**, **H**) and 2 h (**D**, **I**), as well as non-treated controls (**E**, **J**), are shown. The double peak on **J** is most likely due to secondary structure formation by analyzed DNA. LM: Lower alignment marker (50 bp), UM: Upper alignment marker (17,000 bp).
